# Body Mass Index, Lean Mass, and Body Fat Percentage as Mediators of the Relationship between Milk Consumption and Bone Health in Young Adults

**DOI:** 10.3390/nu11102500

**Published:** 2019-10-17

**Authors:** Ana Torres-Costoso, Purificación López-Muñoz, Asunción Ferri-Morales, Elisabeth Bravo-Morales, Vicente Martínez-Vizcaíno, Miriam Garrido-Miguel

**Affiliations:** 1 Facultad de Fisioterapia y Enfermería, Universidad de Castilla-La Mancha, 45071 Toledo, Spain; AnaIsabel.Torres@uclm.es (A.T.-C.); Purificacion.Lopez@uclm.es (P.L.-M.); Asuncion.Ferri@uclm.es (A.F.-M.); Elisabeth.Bravo@uclm.es (E.B.-M.); 2 Centro de Estudios Socio-Sanitarios, Universidad de Castilla-La Mancha, 16071 Cuenca, Spain; Miriam.Garrido@uclm.es; 3 Facultad de Ciencias de la Salud, Universidad Autónoma de Chile, 3467987 Talca, Chile

**Keywords:** milk intake, dairy products, weight status, bone mineral density, bone health, college students, body composition

## Abstract

Identifying environmental factors that influence bone health is crucial for developing effective intervention strategies that maximize peak bone mass. The aim of this study was to estimate the relationship between milk consumption and bone mineral density (BMD) in young adults, and to examine whether this relationship is mediated by body mass index (BMI) and total lean and fat mass. A cross-sectional study involving college students (*n* = 239) from a Spanish public university was performed. Data on milk consumption and anthropometric and body composition variables were collected. The Pearson correlation coefficients among total body BMD, body composition variables, and milk consumption ranged from −0.111 to −1.171, most of them statistically significant (*p* < 0.05). The ANCOVA (analysis of covariance) models showed that those with higher regular milk consumption had less total body BMD than those with lower regular milk consumption (*p* < 0.05), even after controlling for different sets of confounders. In the mediation analysis, BMI and lean and fat mass turned out to act as full mediators of the relationship between regular milk consumption and total body BMD (z = −1.7148, −1.3208, and −1.8549, respectively; *p* ≤ 0.05). In conclusion, milk consumption, per se, does not seem to have a direct effect on bone development, because its association seems to be fully mediated by body composition variables in young adults.

## 1. Introduction

The beginning of adulthood is considered an important opportunity to optimize bone health, since environmental factors play a crucial role in helping skeletal development in this period [1]. Identifying such environmental factors is crucial for developing effective intervention strategies that maximize peak bone mass, defined as the amount of bone attained at the end of skeletal development, and reduce the risk of osteoporosis, falls, and fractures later in life [2,3]. 

Physical activity (PA) has been positively related to bone health outcomes, and physically active youngsters have shown higher levels of lean mass [4], which is a strong predictor of bone mass [5]. In addition, body mass index (BMI) is a well-known environmental determinant of bone health, particularly at the age of peak bone mass due to biomechanical factors that stimulate the osteogenic process [6]. Finally, overweight and obese children and adolescents have higher bone health outcomes than their normal-weight peers [7,8], and this association seems to be explained by their higher levels of both lean and fat mass, which have been shown to be strong predictors for bone mass and, hence, bone strength during growth [9,10]. 

From a nutritional point of view, milk and dairy products are important sources of nutrients, particularly calcium (Ca), which is mainly located in the skeleton and whose main function is to build the bone structure and is responsible for its characteristic rigidity [11]. Ca intake recommendations for European people aged 19–50 years range from 700 mg/day to 1000 mg/day, of which more than 50% come from dairy products [12,13,14]. Thus, an adequate intake from dairy would be 350–500 mg, or 2–3 servings a day. Traditionally, milk and dairy product intake has been associated with normal development of the skeleton, and has played an important role in explaining historical trends of regional differences in human height during the 19th and 20th centuries [15]. However, a recent review reported inconclusive results regarding the relationship between consumption of dairy products and linear growth in children [16], and it is not well known if low-fat milk is better than regular milk at improving skeletal growth. In this sense, to prevent an increased risk of cardiovascular clinical outcomes, current guidelines of some health institutions recommend the consumption of low-fat or fat-free milk [17], although there is little evidence suggesting that the association between milk and dairy products and obesity indicators depends on the type of milk [18]. So far, studies linking milk, bone, and BMI are limited and none have tested whether BMI acts as a confounding or as an intermediate variable in the relationship between milk consumption and bone health.

Thus, the aim of this study was to analyze the relationship between milk consumption and bone mineral density (BMD) in young adults and to examine whether this relationship is mediated by BMI and lean mass and body fat percentage.

## 2. Materials and Methods 

### 2.1. Study Design 

This was a multicenter cross-sectional study aimed at assessing the changes in diet, lifestyle, and cardiovascular risk that occur during university attendance (age 18–30 years old). 

### 2.2. Study Population

A total of 560 first-year university students from the University of Castilla La Mancha were invited to participate in the study, and 360 (64.28%) accepted the invitation. In this report, we used data from a subsample of 239 university students in which BMD (by dual energy x-ray absorptiometry (DXA)) was measured. The young adults included in the data analysis for this study did not differ in age, sex, or parental socioeconomic status from the whole sample of young adults participating in the trial.

### 2.3. Ethics Approval and Consent to Participate

The study protocol was approved by the Clinical Research Ethics Committee of Hospital Virgen de la Luz in Cuenca, REG: 2016jPI1116, and once they were informed verbally and in writing, the participants were asked to sign a consent form as a condition to participate in the study. Because there were no participants aged less than 18 years, which is the legal age in Spain, written informed consent was individually obtained for each participant. The signed informed consent documents were recorded. The Ethics Committee approved the study protocol, including permissions and informed consent documents.

### 2.4. Variables and Measuring Instruments

Anthropometry: Weight was measured twice with the subject barefoot and wearing light clothing using a Seca-770 scale. Height was measured twice with the subject barefoot and upright, with the sagittal midline at the midline of the stadiometer, using a Seca-222 wall-mounted stadiometer. BMI was calculated as weight in kilograms divided by the square of the height in meters (kg/m^2^), using the means of the two determinations of weight and height.

Body composition: The young adults were scanned in the supine position using DXA (Lunar iDXA, GE Medical Systems Lunar, Madison, WI 53718, USA in Cuenca and Hologic Discovery Series QDR, Bedford, USA in Toledo). On the campus of the University of Toledo, the DXA equipment was calibrated using a lumbar spine phantom, following the Hologic guidelines. All the DXA scans were analyzed using Physician’s Viewer, APEX System Software Version 3.1.2. (Bedford, USA). On the campus of the University of Cuenca, the analyses were performed using enCoreTM 2008 software, version 12.30.008. DXA equipment accuracy was checked daily before each scanning session using the GE Lunar calibration phantom, as recommended by the manufacturer. All scans were performed at high resolution by a trained researcher using the same protocol. Total fat mass (%), total lean mass (kg), total body BMD (g/cm^2^), spine BMD (g/cm^2^), and pelvis BMD (g/cm^2^) were calculated for each individual from total analysis of the whole body scan. As two different tools were used to scan, bone variables were normalized for the tool used. In addition, bone variables were normalized by sex for all the analyses.

Milk consumption, dairy products, total calcium, and total energy intake: Milk consumption, dairy products and total energy intake were estimated using a 137-item Food Frequency Questionnaire (FFQ), previously validated in Spain [10]. The FFQ consisted of an incremental scale with nine levels of consumption for each item (never or almost never, 1–3 times per month, once per week, 2–4 times per week, 5–6 times per week, once per day, 2–3 times per day, 4–6 times per day, and more than 6 times per day). Daily food intake was estimated by multiplying the frequency of consumption for each item and the typical portion size specified in the FFQ, based on the latest available information from Spanish food composition tables [19,20]. In the FFQ questionnaire, the standard milk serving size was 200 mL. However, to prevent confusion in the analyses we categorized the consumption as daily intake versus less than daily intake. 

Socioeconomic status (SES): SES was assessed using self-reported occupation and education questions. An index of SES was calculated according to the Spanish Society of Epidemiology scale procedures [19], which categorized family SES in five categories; these five levels were collapsed for our analyses into lower, upper-lower, middle, upper-middle, and upper) [21].

Physical activity (PA): Total amount of physical activity was objectively measured by using GENEActive accelerometers (ActivInsights). Participants wore these devices on their wrists for 7 consecutive days (including nights), set at a fixed frequency of 30.0 Hz, for collecting raw acceleration data measured in “g” for each movement axis (x, y, and z), in order to estimate the participants’ physical activity. We considered as valid measurements those of ≥5 days, including 1 weekend day. For this study, mean total minutes/day of physical activity was estimated [22].

### 2.5. Statistical Analysis

Both statistical (Kolmogorov–Smirnov test) and graphical (normal probability plots) methods were used to examine the fit to a normal distribution for each continuous variable. Then, bivariate correlation coefficients were used to examine the relationship between regular milk and low-fat milk consumption with body composition variables and bone variables.

To test mean differences in total body BMD by regular milk consumption categories, ANCOVA models were calculated. Age and height were covariates in Model 0; age, height, and the average of physical activity were covariates in Model 1; age, height, the average of physical activity, and total calcium intake were covariates in Model 2; and sex, height, the average of physical activity, total calcium intake, and weight were covariates in Model 3. 

We carried out a mediation analysis to determine if BMI, total lean mass, and % total fat mass were mediators in the relationship between regular milk consumption and total body BMD using the PROCESS macro for SPSS (SPSS Inc, Chicago, Illinois). In addition, in order to control the potential confounding role of SES, we conducted a mediation analyses by SES categories. To address these analysis, we used two strategies: (1) nonparametric, as recommended by Preacher and Hayes [23], using a resample procedure of 10,000 bootstrap samples, and (2) parametric using the classical Baron and Kenny steps regression method [24]. To test the statistical significance of the mediation effect in the parametric approach, we used the Sobel test [25]. Statistical analyses were performed with SPSS-IBM (V.24.0 SPSS Inc., Armonk, NY, USA), and the level of significance was set at *p* ≤ 0.05.

## 3. Results

Descriptive characteristics (mean ± SD) of the study sample are shown in Table 1. All variables differed significantly by sex, except age, BMI, regular, total milk and total dairy product consumption, calcium from milk, total calcium intake, and the average of physical activity.

Table 2 shows descriptive characteristics (mean ± SD) of the study sample by regular milk consumption. Daily regular milk intake is significantly related to a lower BMI, % total fat mass, and total body BMD.

Table 3 shows bivariate correlations between regular milk and low-fat milk consumption with body composition variables. BMI and total body BMD were significant and negatively associated with regular milk, but not with low-fat milk and total dairy products (*p* < 0.05). In addition, spine and hip BMD did not significant correlate with milk and total dairy products consumption.

Table 4 shows the mean-adjusted differences in total body BMD by regular milk consumption categories, after controlling for potential confounders. Young adults with high milk consumption showed significantly lower total body BMD than their peers after controlling for age and height (Model 1), for the average of physical activity (Model 1), and for total calcium intake (Model 2) (*p* < 0.05). However, the differences did not reach statistical significance when controlling for weight (Model 3).

**Figure 1:**
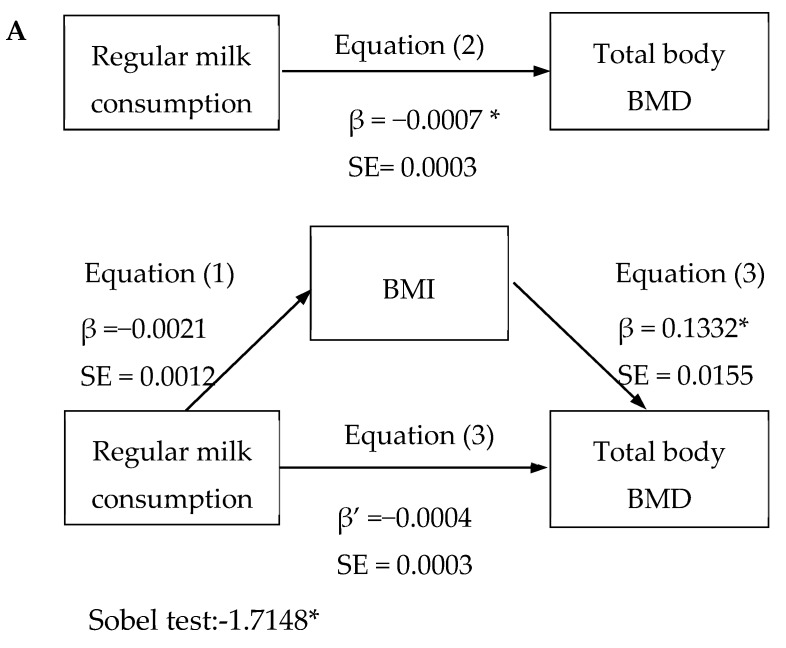

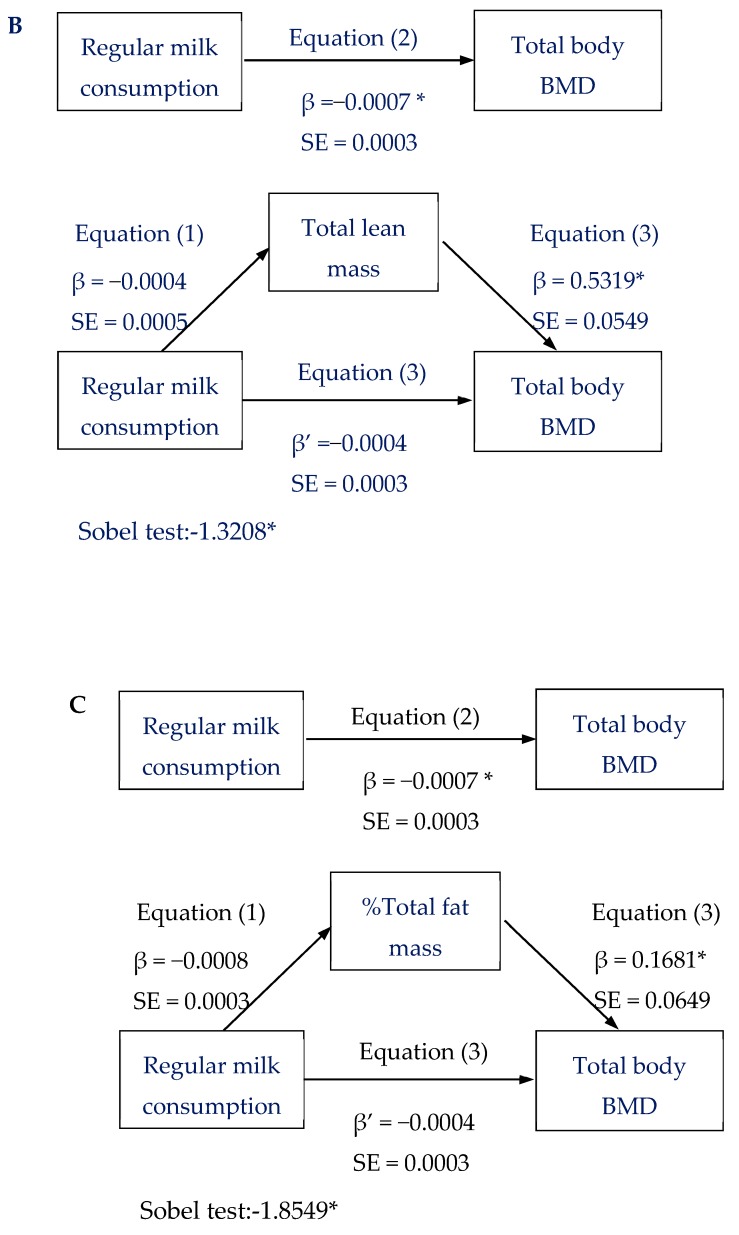
(**A**–**C**). Body mass index (BMI), total lean mass, and % total fat mass mediation models of the relationship between regular milk consumption and bone mineral density (BMD). SE (standard error of beta estimate). * *p* < 0.05.

Figure 1A displays the simple mediation analysis, which showed that regular milk consumption indirectly influences total body BMD through its association with BMI. In the first regression equation, the relationship between regular milk consumption and BMI was negative (b = −0.0021; *p* > 0.05). In the second regression equation, the relationship between regular milk consumption and total body BMD was positive (b = 0.0007; *p* < 0.05). In the third regression equation, the relationship between BMI and total body BMD was positive (b = 0.1332; *p* < 0.05), and the relationship between regular milk consumption and total body BMD was attenuated when the mediator was included in the regression model (b = −0.004; *p* > 0.05). Thus, total BMI acted as a full mediator of the relationship between regular milk consumption and total body BMD, as tested by the Sobel test for indirect effect (z = 4.43; *p* < 0.001). The percentage of total effect mediated by total lean mass was 26.8%. When we tested the mediator role of total lean mass and % total fat mass in the relationship between regular milk consumption and total body BMD, the results were similar to those described using BMI as a mediator (Figure 1B,C). Additionally, we performed the analysis stratifying by gender and SES, and the magnitude of the coefficients estimates was similar, although, because of the sample size reduction, the significance disappeared (Figure S1, S2, S3, and S4 in Supplementary Materials).

## 4. Discussion

The present study shows that young adults with higher regular milk consumption have less total body BMD than those with lower regular milk consumption; however, these differences disappeared after controlling for relevant sets of confounders, including age, height, and weight. In addition, BMI, total lean mass, and body fat percentage act as total mediators in the association between regular milk consumption and total body BMD.

The National Osteoporosis Foundation recommends dairy product consumption in order to improve peak bone mass [26]. Despite the recognized advantages of milk as a good source of key bone-specific nutrients (calcium, phosphorous, and protein) and a balanced diet, a substantial proportion of young people do not meet consumption recommendations. In the same line, our results show that, although the total calcium intake is adequate, only 23.19% comes from milk.

A growing body of evidence indicates that dairy products, and particularly milk, are important diet components for linear growth and bone health during development [3,27,28]. However, in our study, we observed that in young adults the daily milk consumption showed a negative relationship with total body BMD after controlling for age and height (Model 0), physical activity (Model 1), and total calcium intake (Model 2) than their peers with less than the daily consumption. However, these differences steadily disappeared once we controlled for body weight (Model 3), suggesting that the significant differences observed in Models 0, 1, and 2 might be explained by the effect of weight status. Moreover, this association disappeared when we stratified by SES, probably because, although there is a lack of evidence of a relationship between milk intake and SES [29], we cannot rule out the possibility that this may be due to the lack of statistical power because of the small sample size.

Currently, there is consistent evidence about the important role of weight-dependent loading of the skeleton in establishing and maintaining bone mass and strength during development. The direct pathway for weight to influence bone is thought to be mechanical loading [30,31]. In accordance with these data, our findings support the positive association between BMI and bone outcomes. 

The influence of milk on weight status during development is controversial. Previous results in children have suggested that excess dairy protein intake may enhance weight gain through the induction of insulin and insulin-like growth factor-1 [32]. On the contrary, in accordance with our data, other studies have reported that milk consumption is inversely related with the risk of being overweight and obesity during growth [33,34]. Furthermore, even though several national guidelines specifically encourage low-fat dairy product consumption [27], our results indicate no relationship between fat-free milk and total body BMD and a positive relationship between fat-free milk and BMI.

In summary, our results show a significant and negative relationship between regular milk intake, weight status, and total body BMD but show a positive relationship between BMI and total body BMD. Thus, our mediation analyses suggest that acceptable regular milk consumption is not enough to optimize bone health, because BMI plays a pivotal role in this association. Additionally, young adults with a higher regular milk consumption showed lower BMI and consequently lower total body BMD. Similar to these results, total lean mass and total fat mass percentage showed a mediator role in this association, which reinforces the evidence regarding the relationship between mechanical stimulation and bone outcomes, as explains the mechanostat theory that has argued that bones adapt their resistance to the mechanical loads placed on them [35]. Overall, the estimates were similar in boys than in girls, although when stratified by gender the results’ statistical significance disappeared. 

Our study has some limitations that should be stated. First, the analysis had a cross-sectional design; therefore, we cannot make cause-effect inferences. Second, we cannot generalize our results owing to the many factors that influence bone health (genetic, nutritional, and environmental). Third, the whole body variable sample that was studied included only college students, so caution is necessary when making inferences about other age ranges. Fourth, self-reported consumption of dairy products through the 137-item FFQ may be biased because the questionnaire used for estimating the participants’ intake did not differentiated between fortified and non-fortified milk consumption; thus, we cannot examine its influence on bone health. Fifth, although the total body less head is a recommended assessment site for the monitoring of bone health [36], in our sample this variable was not available for the analyses. Sixth, because the mean dairy intake is not sufficient to comply with European recommendations [27], our mediation analysis is limited to this population’s dairy intake; we cannot know if our conclusions would be maintained in the case of populations that comply with the recommendations of dairy intake. Finally, other products that were not controlled may have influenced the relationship between milk consumption and bone outcomes.

## 5. Conclusions

Our data are relevant from a clinical perspective, because they disclose that body composition variables play a pivotal role in the relationship between milk consumption and bone health. Milk consumption, per se, does not seem to have a direct effect because its association with bone development seems to be mediated by weight status and lean mass and fat mass percentage in young adults.

## Figures and Tables

**Table 1:** Sample characteristics

	All (239)	Boys (79)	Girls (160)	*p*
**Age (years)**	21.44 ± 3.36	21.31 ± 2.52	21.51 ± 3.71	n.s.
**Weight (kg)**	64.33 ± 2.12	70.75 ± 10.62	61.15 ± 11.57	**<0.001**
**Height (cm)**	166.26 ± 8.36	174.11 ± 6.99	162.44 ± 5.96	**<0.001**
**BMI (kg/m^2^)**	23.17 ± 3.60	23.24 ± 2.64	23.14 ± 3.99	n.s.
**%Total fat mass**	26.66 ± 10.00	18.85 ± 6.85	30.68 ± 8.95	**<0.001**
**Total lean mass (Kg)**	43.04 ± 9.31	53.54 ± 6.83	37.82 ± 4.96	**<0.001**
**Total body BMD (g/cm^2^)**	1.131 ± 0.111	1.197 ± 0.121	1.099 ± 0.092	**<0.001**
**Spine BMD (g/cm^2^)**	1.045 ± 0.129	1.075 ± 0.152	1.030 ± 0.113	**0.010**
**Pelvis BMD (g/cm^2^)**	1.061 ± 0.147	1.141 ± 0.159	1.022 ± 0.123	**<0.001**
**Regular milk (mL/d)**	87.27 ± 199.24	127.38 ± 233.68	67.97 ± 177.95	n.s.
**Fat-free milk (mL/d)**	37.58 ± 121.42	6.47 ± 30.35	52.55 ± 144.04	**<0.001**
**Total milk consumption (mL/d)**	120.18 ± 200.22	148.34 ± 250.67	104.88 ± 189.67	n.s.
**Total dairy products (g/d)**	392.26 ± 277.40	426.97 ± 279.87	373.40 ± 274.81	n.s.
**Total energy intake (Kcal)**	2342.78 ± 769.94	2459.26 ± 805.18	2340.60 ± 747.95	**0.038**
**Ca from milk (%)**	23.19 ± 14.92	24.82 ± 16.07	22.30 ± 14.22	n.s.
**Ca (mg/dL)**	1219.77 ± 555.30	1241.32 ± 562.64	1208.07 ± 542.65	n.s.
**Average PA (min/d)**	223.17 ± 65.33	221.48 ± 76.91	223.69 ± 67.68	n.s.
**SES (%)**				
**Low**	28.3	30.8	27.0	**0.048**
**Medium**	46.6	52.1	43.7	
**High**	25.1	17.1	29.3	

Results are shown as mean ± SD. Bold values indicate *p* < 0.05; n.s., non-significant. Abbreviations: BMI, body mass index; BMD, bone mineral density; PA, physical activity, SES, socioeconomic status. T student tests (continuous variables) or chi squared tests (categorical variables).

**Table 2:** Sample characteristics by regular milk consumption.

	Less than Daily Intake (187)	Daily Intake (52)	*p*
**Age (years)**	21.17 ± 3.34	20.74 ± 2.12	n.s.
**Weight (kg)**	65.92 ± 12.70	63.27 ± 10.68	n.s.
**Height (cm)**	166.99 ± 8.65	168.45 ± 8.38	n.s.
**BMI (kg/m^2^)**	23.55 ± 3.78	22.21 ± 2.79	**0.006**
**% Total fat mass**	27.56 ± 10.12	23.24 ± 9.07	**0.002**
**Total lean mass (Kg)**	42.44 ± 9.09	44.86 ± 9.68	n.s.
**Total body BMD (g/cm^2^)**	0.050 ± 0.999	−0.137 ± 0.902	**0.05**
**Spine BMD (g/cm^2^)**	0.039 ± 1.014	−0.134 ± 0.865	n.s.
**Pelvis BMD (g/cm^2^)**	0.042 ± 0.987	−0.105 ± 0.940	n.s.
**Total milk consumption (mL/d)**	55.77 ± 123.17	332.73 ± 203.98	**<0.001**
**Total dairy products (g/d)**	343.44 ± 238.60	541.63 ± 252.53	**<0.001**
**Total energy intake (Kcal)**	2630.55 ± 1270.72	3119.23 ± 1191.15	**0.004**
**Average PA (min/d)**	224.27 ± 72.56	220.25 ± 56.59	n.s.
**SES (%)**			
**Low**	23.8	4.8	n.s.
**Medium**	35.8	9.9	
**High**	20.2	5.4	

Results are shown as mean ± SD. Bold values indicate *p* < 0.05; n.s., non-significant. Abbreviations: BMI, body mass index; BMD, bone mineral density; PA, physical activity, SES, socioeconomic status. T student tests (continuous variables) or chi squared tests (categorical variables).

**Table 3:** Bivariate correlations among body composition variables and milk consumption.

	Regular Milk	Fat-Free Milk	Total Milk Consumption	Total Dairy Products	Total Body BMD	Spine BMD	Pelvis BMD	BMI	% Total Fat Mass	Total Lean Mass	Average PA	Total Energy Intake
**Regular milk**	-	−0.118 *	0.860 **	0.551 **	−0.133 *	−0.106	−0.101	−0.111 *	−1.171 **	0.078	0.069	0.138 *
**Fat-free milk**			0.406 **	0.274 **	0.112	0.145 *	0.056	0.141 **	0.159 **	−0.104	−0.065	−0.059
**Total milk consumption**			-	0.648 **	−0.058	−0.017	0.391	−0.030	−0.070	0.014	−0.098	0.325 **
**Total dairy products**				-	−0.065	−0.043	−0.079	−0.047	−0.133 *	0.012	0.081	0.472 *
**Total body BMD**						0.783 *	0.730 *	0.498 **	0.142 **	0.323 **	0.013	−0.079
**Spine BMD**						-	0.670 *	0.477 *	0.175 *	0.255 **	−0.067	−0.075
**Pelvis BMD**							-	0.356 *	0.082	0.271 *	−0.030	−1.132 *
**BMI**									0.493 *	0.323 *	0.057	−0.127 *
**%Total fat mass**									-	−0.496 **	−0.022	−0.166 *
**Total lean mass**										-	0.062	−0.061
**Average PA**											-	0.024

Data are presented in the correlation coefficient R. * *p* < 0.05, ** *p* < 0.01. Abbreviations: BMI, body mass index; BMD, bone mineral density; PA, physical activity.

**Table 4:** ANCOVA models comparing means of bone mineral density (BMD, g·cm^−2^) by categories of regular milk consumption.

		Total Body BMD			
**Regular Milk Consumption**	***n***	**Model 0**	**Model 1**	**Model 2**	**Model 3**
****Less than daily intake****	185	0.07 (0.07)	0.15 (0.95)	0.17 (0.95)	0.11 (0.08)
****Daily intake****	51	−0.24 (0.13)	−0.43 (0.20)	−0.50 (0.21)	−0.24 (0.18)
***p***		**0.042**	**0.011**	**0.005**	0.081

Results are shown as mean ± SD. Bold values indicate *p* < 0.05. Model 0: age + height; Model 1: Model 0 + average of physical activity; Model 2: Model 1 + calcium; Model 3: Model 2 + weight. *n*.s., non-significant.

## Supplementary Materials

The following are available online at https://www.mdpi.com/2072-6643/11/10/2500/s1: Figure S1A,B; body mass index (BMI) mediation models of the relationship between regular milk consumption with bone mineral density (BMD) by sex. SE (stander error of beta estimate),* *p* < 0.05. Figure S2A,B; total lean mass mediation models of the relationship between regular milk consumption with bone mineral density (BMD) by sex. SE (standard error of beta estimate), * *p* < 0.05. Figure S3A,B; %Fat mass mediation models of the relationship between regular milk consumption with bone mineral density (BMD) by sex. SE (standard error of beta estimate), * *p* < 0.05. Figure S4A,B,C; body mass index (BMI) mediation models of the relationship between regular milk consumption with bone mineral density (BMD) by socioeconomic status (SES), adjusted by total energy intake. SE (standard error of beta estimate), * *p*<0.05.
