# Supplementary material for: Body Mass Index, Lean Mass, and Body Fat Percentage as Mediators of the Relationship between Milk Consumption and Bone Health in Young Adults
Source: Nutrients. 2019 Oct 17;11(10):2500. doi: 10.3390/nu11102500 (PMC6835529; doi:10.3390/nu11102500)

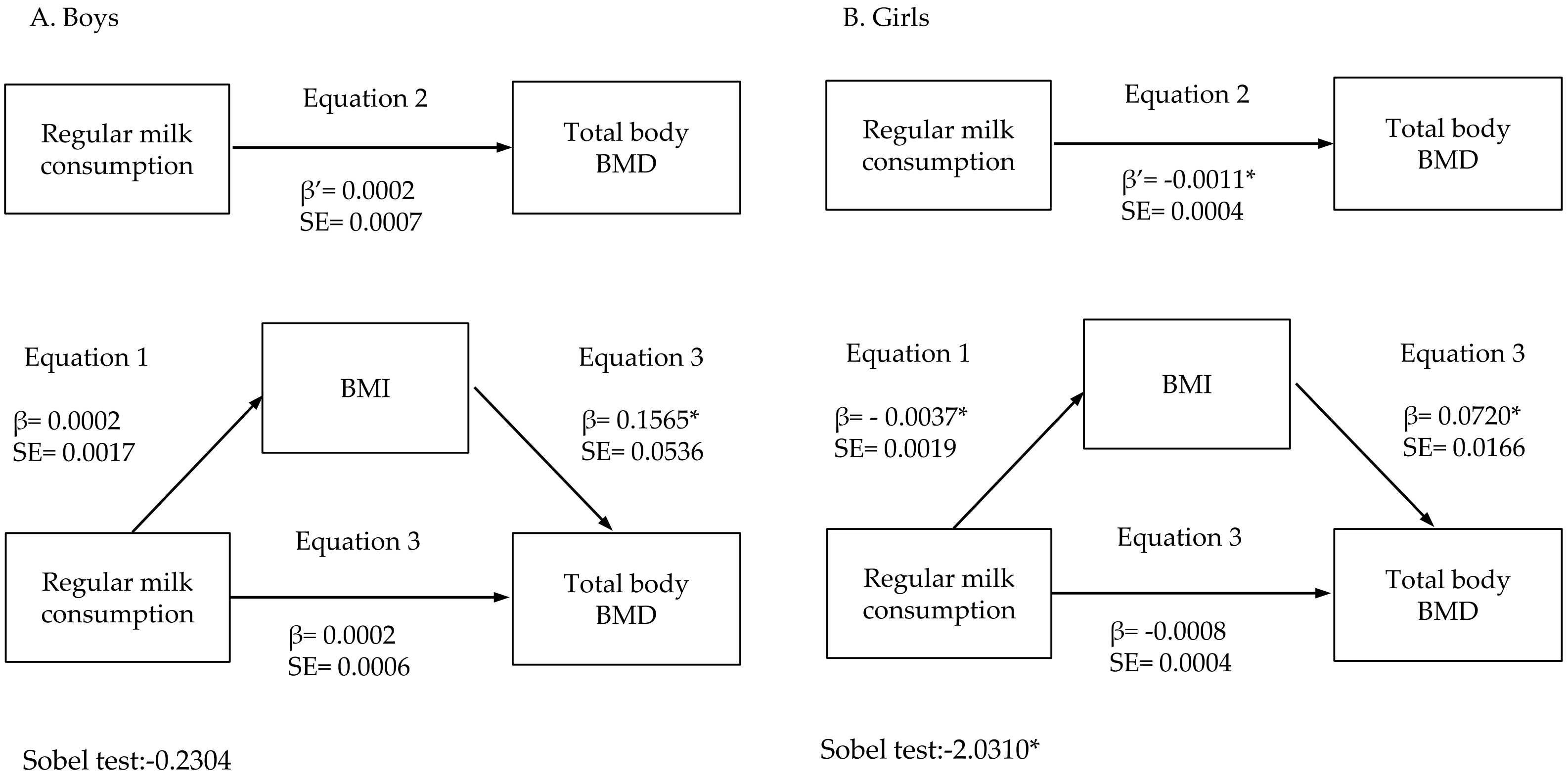

Figure S1. A, B. Body mass index (BMI) mediation models of the relationship between regular milk consumption with bone mineral density (BMD) by sex. SE (standar error of beta estimate). *p<0.05

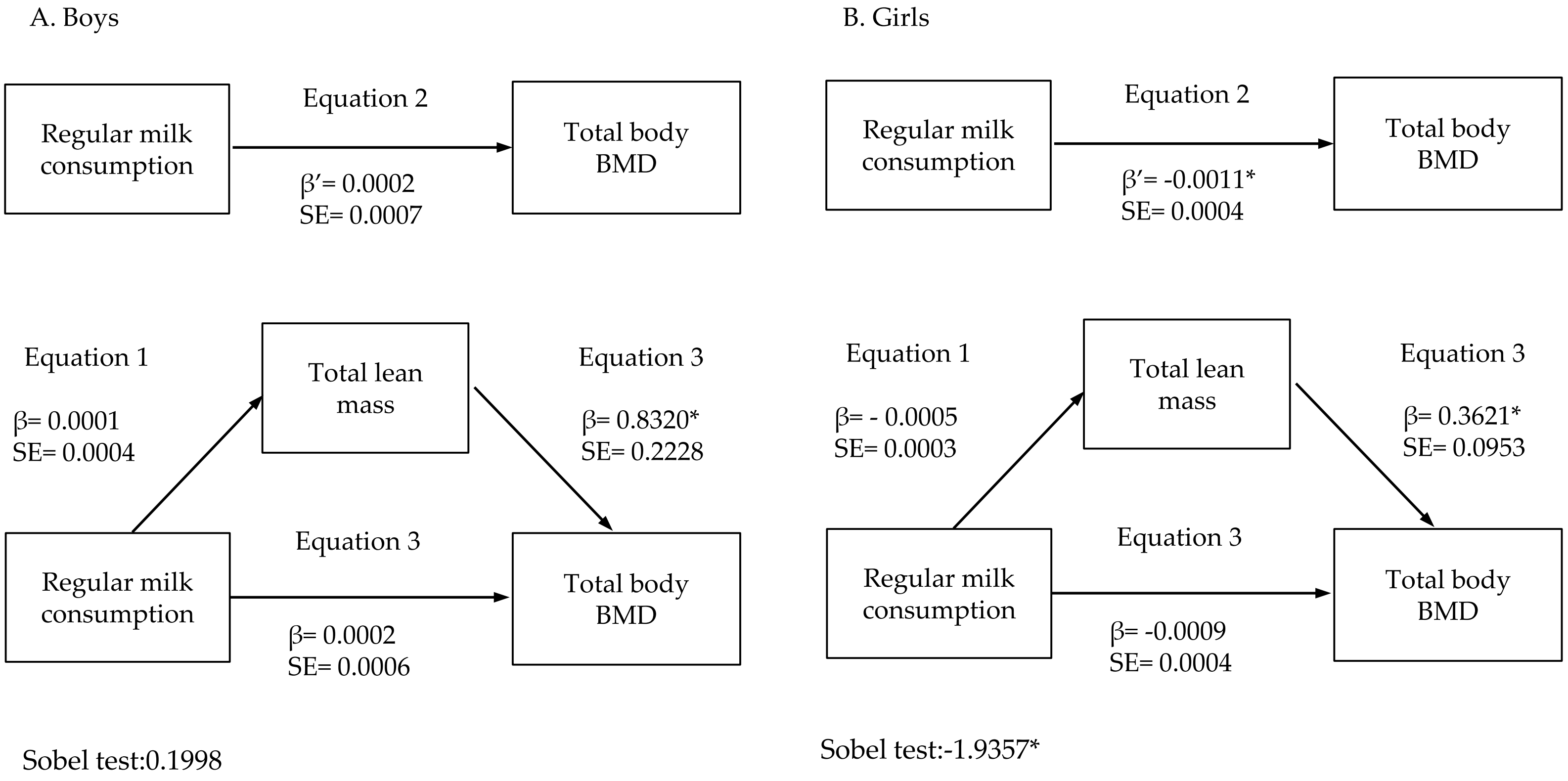

Figure S2. A, B. Total lean mass mediation models of the relationship between regular milk consumption with bone mineral density (BMD) by sex. SE (standar error of beta estimate). *p<0.05

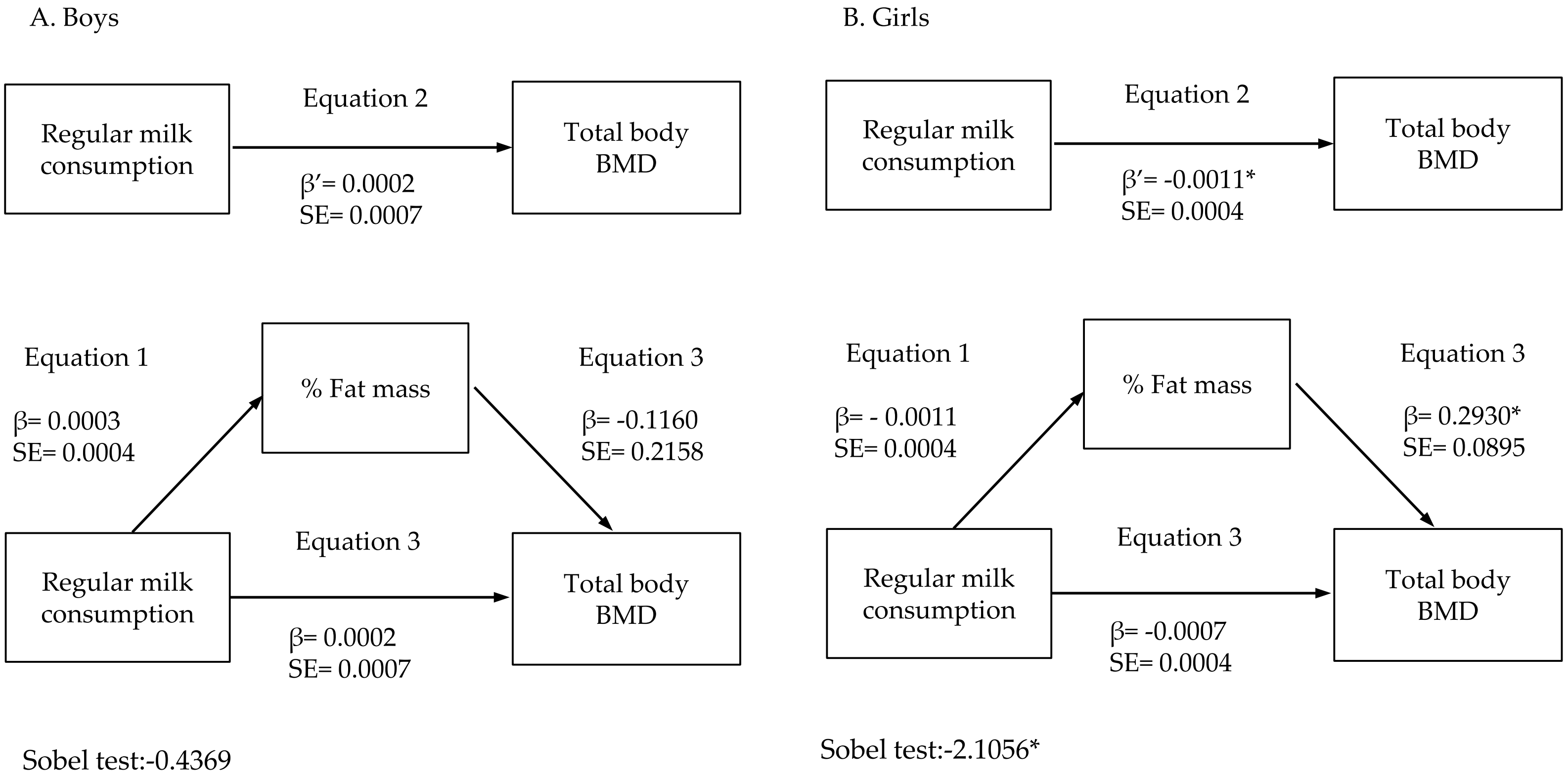

Figure S3. A, B. % Fat mass mediation models of the relationship between regular milk consumption with bone mineral density (BMD) by sex. SE (standar error of beta estimate). *p<0.05

Figure 4. A, B, C. Body mass index (BMI) mediation models of the relationship between regular milk consumption with bone mineral density (BMD) by socioecomic status (SES) adjusted by total energy intake . SE (standar error of beta estimate)
*p<0.05

A. Low SES

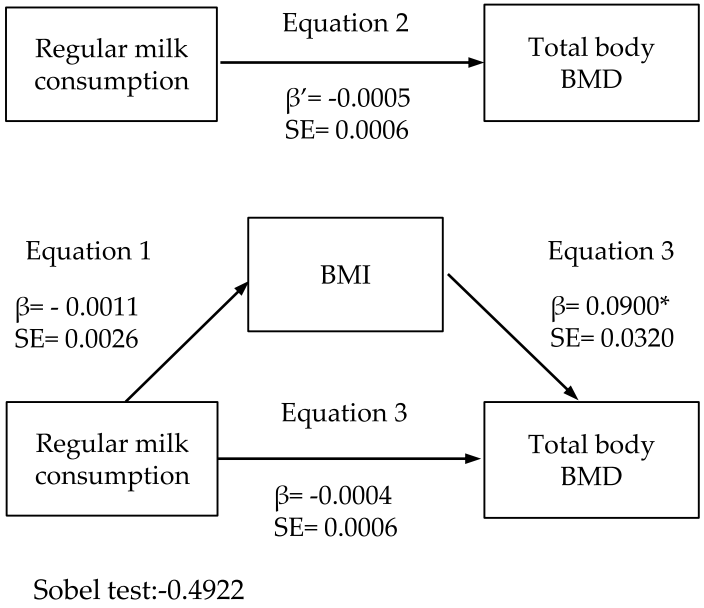

B. Medium SES

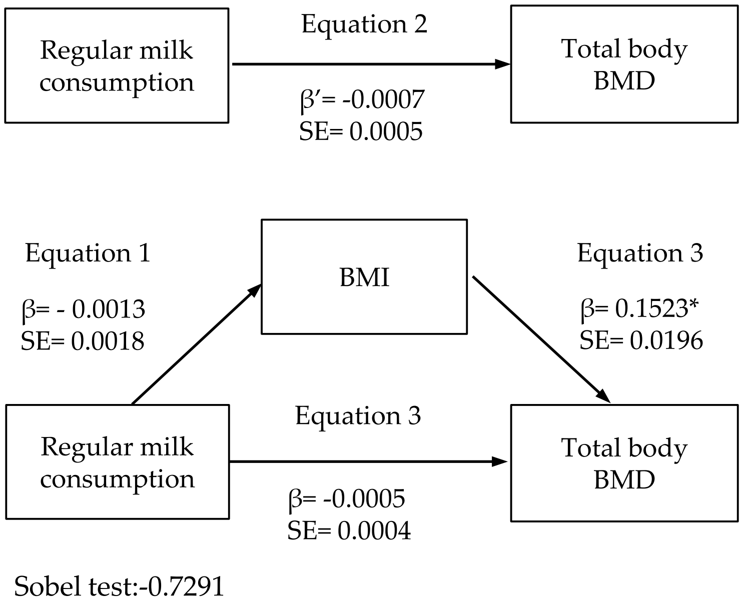

C. High SES

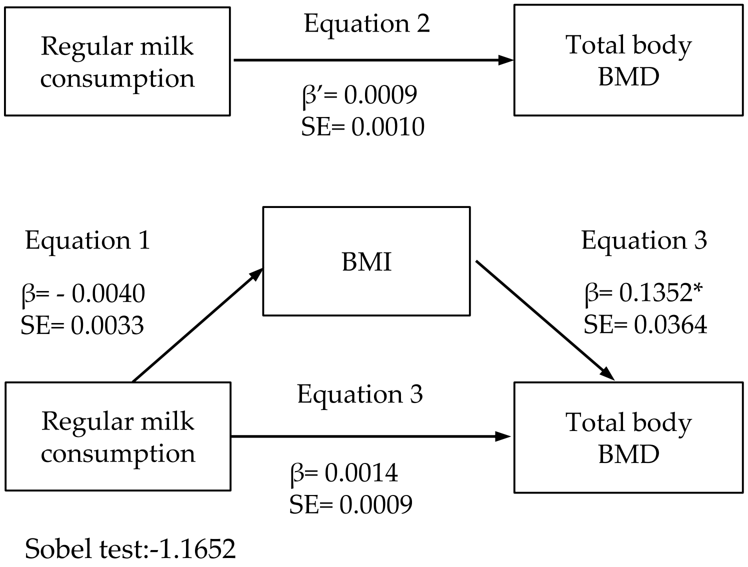

Supplement: Supplementary file 1 [file nutrients-11-02500-s001.pdf]
